# VISUALIZING EATING EXPERIENCES IN DEMENTIA: USING MASLOW’S HIERARCHY OF NEEDS AND THE MUNSELL COLOR SYSTEM

**DOI:** 10.1093/geroni/igae098.3086

**Published:** 2024-12-31

**Authors:** Zih-Ling Wang, Jenna McHale

**Affiliations:** University of Washington, Seattle, Washington, United States; University of Washington, Seattle, Washington, United States

## Abstract

Eating experiences among individuals with dementia represent a multifaceted, subjective, and unique life journey. Despite their complexity, studies analyzing these experiences are limited. This study aims to explore the various domains that shape the trajectory of eating experiences in individuals living with dementia, adapting the Maslow’s Hierarchy of Needs five-stage model and Munsell Color System to map these experiences visually. This presentation introduces an innovative model for visualizing the antecedents of eating experiences. It proposes strategies to enhance positive outcomes and nutritional health in individuals with dementia throughout their experience trajectory. Eating experiences are described across five domains: Aesthetic, Esteem, Sociocultural and Belonging, Safety, and Physiological and Biological. As dementia progresses, increased dependency, the domains measured by degree inward towards the center, can significantly affect these eating experiences. These domains are situated by their degree, with each domain independently fluctuating in size and shape based on the degree of maintained personhood as dependency needs shift. Minimal variation within these domains is essential for optimizing the eating experience. Each domain-related experience is subject to fluctuation over time, influenced by both external and internal life events. The trajectory’s shape is a visual representation of changes in experience over time. By identifying positive and negative cases within the eating experiences, this presentation illustrates how deficiencies in specific domains can lead to negative eating experiences. Additionally, the presentation offers health professionals a method to tailor care strategies for enhancing eating experiences by interpreting current and trend domain mappings.

